# Organ-on-a-Chip systems for new drugs development

**DOI:** 10.5599/admet.942

**Published:** 2021-03-22

**Authors:** Ronny Vargas, Andrea Egurbide-Sifre, Laura Medina

**Affiliations:** 1 Industrial Pharmacy Department, Faculty of Pharmacy, University of Costa Rica 11501-2060, San José, Costa Rica; 2 Faculty of Pharmacy and Food Sciences, University of Barcelona, Av. Joan XXIII, 27-1, 08028, Barcelona, Spain

**Keywords:** Drug Discovery, Tissue Engineering, Cell Culture, Preclinical models, Organoids, Lab-On-A-Chip Devices

## Abstract

Research on alternatives to the use of animal models and cell cultures has led to the creation of organ-on-a-chip systems, in which organs and their physiological reactions to the presence of external stimuli are simulated. These systems could even replace the use of human beings as subjects for the study of drugs in clinical phases and have an impact on personalized therapies. Organ-on-a-chip technology present higher potential than traditional cell cultures for an appropriate prediction of functional impairments, appearance of adverse effects, the pharmacokinetic and toxicological profile and the efficacy of a drug. This potential is given by the possibility of placing different cell lines in a three-dimensional-arranged polymer piece and simulating and controlling specific conditions. Thus, the normal functioning of an organ, tissue, barrier, or physiological phenomenon can be simulated, as well as the interrelation between different systems. Furthermore, this alternative allows the study of physiological and pathophysiological processes. Its design combines different disciplines such as materials engineering, cell cultures, microfluidics and physiology, among others. This work presents the main considerations of OoC systems, the materials, methods and cell lines used for their design, and the conditions required for their proper functioning. Examples of applications and main challenges for the development of more robust systems are shown. This non-systematic review is intended to be a reference framework that facilitates research focused on the development of new OoC systems, as well as their use as alternatives in pharmacological, pharmacokinetic and toxicological studies.

## Introduction

The development of new drugs is an expensive process that presents several challenges in preclinical and clinical studies [1,2], where a 59 % of the medicines that initiate a clinical study go into phase II, just a 21 % starts phase III, and merely a 10 % reach approval by the regulatory authority. The appearance of adverse effects is one of the main reasons for drug failure, becoming one of the biggest challenges and weaknesses presented by preclinical models as predictors of clinical performance [2–5]. However, clinical failures are not just the result of safety-related situations, but are also linked to the lack of clinical efficacy [6]. The non-detection of side effects can also become an issue for a drug that has already passed the clinical phase. The non-adequate determination of adverse effects can lead to market recalls. These unwanted events are mainly liver- or heart-related, due to the effect of metabolite generation [5,7].

Animal models applied in preclinical phases are often unrepresentative to the human body, thus failing in the prediction of the efficacy and safety of drugs [1,3,4,8,9]. However, clinical failures might be mitigated by adding models with a superior predictor capacity in preclinical phases [10]. The use of traditional two-dimensional (2D) cell cultures has made great contributions to medicine so far, and it has led to reduce the use of laboratory animals. However, these systems do not allow the study of tissue’s microenvironment or the complex interrelationships between different tissues and organs of the human body. Moreover, the study of isolated cells without a systematic functional evaluation presents the risk of ignoring structural alterations that may have an impact on the organ’s function, even if those isolated cells remained viable [1,10].

In recent years, the Organ-on-a-Chip (OoC) technology has emerged as an alternative in drug development, as it aims to reflect the environmental, functional, and interrelationship characteristics of organs and tissues [2,11]. The concept of reproducing organic and physiological functions of the human body by using cells within a microfluidic chip was first published in 2004, when Shuler *et al.* showed a cell culture that exemplified the interaction between the lung and the liver on a square-inch silicone chip. The term Organ-on-a-Chip was adopted in 2010 [2,7,12,13].

Compared with traditional models, this technique provides improved information about cells mechanical properties, morphology, and differentiation, among others. OoC improve the determination of cell responses, genetic expressions, and cell function. These systems can have an impact on the candidates selection and evaluation for the development of new medicines, as they allow gaining information about metabolites secretion performed by certain cells when interacting with drugs [1]. Furthermore, OoC have the potential to reduce and supplement the use of animals, cell models, and even humans in new drugs development [7].

OoC systems offer a better understanding of pathophysiology, allowing new drugs design into specific mechanisms and promote therapeutic development for pathologies such as cancer, neurological disorders, and orphan diseases [2]. Choi *et al*. used a tridimensional (3D) cancer model for the selection of a most effective drug against lung cancer cells [14]. Liu *et al*. studied brain metastasis with a multi-organ chip that showed functional barrier characteristics [15]. During the COVID-19 pandemic caused by the SARS-CoV-2 in 2020, and in the middle of a global race to slow down the progression of the disease, OoCs were applied in the research for treatments with fast and reliable results on the preclinical scale [16].

The development of these models brought to the emergence of an industry with more than 28 companies founded in 7 years [7,17,18]. Hundreds of publications are generated each year regarding this topic, which rises the interest of companies carrying out basic research in the field of drugs and also in other areas such as cosmetics, food and medical devices [8,11,19–22].

Some of the synonyms under which OoC can be found in the literature are the following ones: lab-on-a-chip [17], organoids-on-chip [23], organ-on-a-plate[6], tissue-on-chip [9], microphysiological system (MPS)[6], and MPS-based organ model [24]. This work is intended to be a reference framework that facilitates research focused on the development of new OoC systems, as well as their use as alternative in pharmacological, pharmacokinetic and toxicological studies with the aim of making them become a more powerful, accurate and used research tool in drug development.

## OoC basic features

This field of study emerges as a combination of cellular biology, cell culture engineering, microsystem engineering, microfluidic studies, and materials engineering. This complexity of disciplines allows to create microenvironments with precise mechanical, and structural control, providing the ability to recreate concentration gradients, and nutrients and metabolites flows [2,3,7,25–31]. Therefore, they are versatile and modifiable systems with the aim of showing an adequate histological composition that is suitable for live evaluation, which cannot be done by using animal models [32].

As an interdisciplinary field, a broader understanding of the OoC technology requires knowledge in each of the sciences that are part of it. Since the evolution of these devices involves technology advances in each of these areas, it faces the same difficulties as they do [18], especially with the objective of mass production and sustainable implementation of OoC. Some of these challenges are discussed later in this document, although the authors recommend a further study of the basic principles and state of the art of the abovementioned scientific fields. Detailed reviews focused on each of these research areas can be consulted elsewhere [33–41].

**Figure 1.**
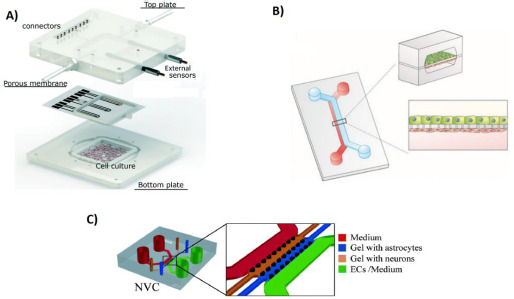
Examples of OoC devices. **A)** Three-layer chip with a cell culture receptacle on the bottom plate, and multiple channel connectors for feed and signal control on the top plate. Adapted from [46] with permission from The Royal Society of Chemistry. Copyright (2018) **B)** A Two-channel OoC for the simulation of an epithelial tissue. Reproduced from [47] under terms of the Creative Commons Attribution License. **C)** OoC model for the simulation of the blood-brain barrier with gel suspended astrocytes and neurons. *Reproduced from [48] with permission from The Royal Society of Chemistry. Copyright (2017)*.

The spatial configuration of each OoC depends on its design and desired function, however, most of them show the general outlines presented in Figure 1; they are constituted by a physical compartment for cell confinement and several microfluidic channels, which are used for the administration and transport of substances and measurable signals. In their integrated compartment OoCs allow to study, measure and control cell behavior, as well as the response of the target tissue microenvironment in front of certain stimuli [2]. It is also possible to integrate secondary tissues in the OoC, which cannot be performed in traditional cellular models [5].

The OoC can reproduce particular conditions, such as the presence of specific pathologies, age and sex differences, or metabolic modifications [3,7,8,15,42–44]. The ability of OoC systems to mimic the functionality of an organ allows to visualize the impact of a condition on the organ’s function. For example, for the study of a viral infection at kidney level, a traditional 2D culture would be able to demonstrate the virus replication but its impact on the kidney’s function would not be proved unless the 2D culture was coupled to an OoC system capable of mimicking glomerular filtration [45].

OoC systems are intended to be a useful tool to reduce the number of false positives and false negatives in the preclinical evaluation of potential drug prototypes. Therefore, they might be able to raise the number of candidates that successfully complete clinical trials [6]. Moreover, these systems have fewer ethical or animal welfare concerns than traditional animal models [49,50]. OoC can facilitate the design and validation of drugs that aim specific cellular and molecular targets [18,44], and could be used to establish the pharmacokinetic profile of drugs [51]. Table 1 shows some of the main characteristics of existing preclinical models for a comparative benefits-drawbacks evaluation.

## OoC functional characteristics

The design of OoC involves reverse engineering of living organs [42,52], which requires an in-depth study of the organs and their functions to develop systems more physiologically relevant than current *in vitro* models. This has promoted the physio-pathological study of multi-organ interrelationships [31,42,44,49,53]. The main benefits of OoC arise from their ability to mimic three key organ aspects in their physiological function [7,54], which are:

1. Interfaces that present barrier functions composed of multiple tissues and vasculature.
2. Organization at a parenchymal level (i.e. the presence of different tissue substructures within the same organ).
3. The interaction between different organs.

Additionally, to be adaptable to HTS, OoC systems require the incorporation of sensors and communication channels [7,27,28] for real-time data collection and analysis [42].

### Reproduction of barrier or interface properties

Non-homogeneous cell mixtures contain variations in shape, arrangement and interaction through interfaces of different cells, which limits the use of 2D cell cultures [7]. Traditionally, epithelia used to be reproduced *in vitro* as several cell layers on a surface. However, the evolution of OoC production systems, and the ability to diversify 3D structures, allow a more accurate correspondence with the physiological and anatomic-functional conditions of epithelial barriers [47]. OoC microfluidic channels can incorporate polymeric membranes to model tissue interfaces, simulate barrier properties and, in some cases, they can even include programmed mechanical instruments [7,50,55].

The possibility of establishing a 3D vascular structure enables to extend the complexity and representativeness of the systems mimicked in an OoC. This is especially valuable with regard to the targeting of nutrients and exogenous substances if compared to 2D tissues, where transport occurs by simple diffusion [55]. Furthermore, OoCs have the ability to include dynamic factors that simulate complex biological events [7,56].

On one hand, the simulation of the physiological interface can be performed in the chip’s primary physical structure itself or by controlling the mobility of hydrogels through the channels of this polymeric structure [7]. Current printing techniques allow complex 3D structures to simulate vascularized tissues and to modulate permeability through the creation of nano-porosities [7,42,57,58]. Therefore, it is possible to provide mechanical stability to functional cell cultures and to generate a network designed for the transport of substances and to simulate specific desired (even pathogenic) organ-organ interaction conditions [7,26]. On the other hand, the use of hydrogels (e.g. gelatine, fibrin or collagen) within the chip channels generates hydrophilic networks that provide the system with slight selective permeability [43,59,60]. The mimicking of several epithelial structures such as blood vessels, alveoli and the blood-brain barrier have been achieved by using OoCs [61].

### Reproduction of parenchymal tissue

In a living organism, cells are specifically organized according to tissue’s function [2,7,43,62]. The arrangement of these cells is one of the main features to consider for the physiological simulation outside the native environment [8,31,42,63]. While membranes and barriers can be simulated in both 2D and 3D cultures, complex parenchymal structures cannot be mimicked in a 2D culture [1,7,8,28,43,63]. Furthermore, gene expression of cells has been observed to be greater in 3D tissues compared to 2D cell cultures [64].

Considering two models of cardiotoxicity assessment, an *in vitro* 2D model enables the evaluation of the cardiomyocytes viability in the presence of a drug but it cannot assess the functionality of the cells. Nevertheless, the effect that this drug may produce in the contraction capacity of cardiomyocytes could be observed by using a 3D arrangement. An adverse reaction, such as arrhythmia, might not be detected in the 2D model and would become a potential issue on further clinical phases, but it could be noticed employing an OoC that evaluates changes in the 3D accommodation and the proper functionality of the organ [44,65].

### Reproduction of the interaction between organs

**Figure 2.**
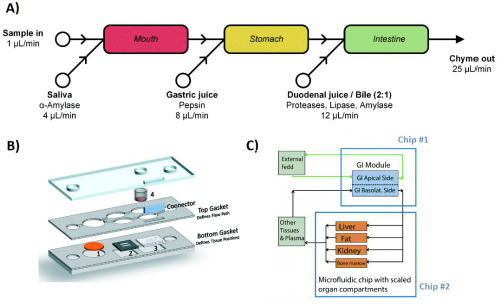
Organ interaction in OoC. **A)** Schematic representation of miniaturized digestive system. **B)** Heart–liver body-on-a-chip with a liver module, cardiomyocytes and a skin module on a single chip. **C)** Body-on-a-chip simulation with gastrointestinal tract and liver tissue with two coupled chips. *Adapted respectively from [69], [67], and [68] with permission from The Royal Society of Chemistry. Copyright (2019, 2020 & 2014)*

Living tissues are highly integrated, they are not isolated, in such a way that the effects in one tissue can impact other tissues [66]. Intercommunication between different organs plays an important role in the safety of a drug and, thus, in the cost-benefit ratio of the clinical studies [42]. This inter-organ communication is absent in most traditional cell cultures, however, OoCs allow the coupling of different organs according to their physiological disposition [42,44,51], either on a chip with different cavities or by interconnecting different chips [7,42], as shown in Figure 2. The mimicking of this organ-organ interaction in OoC leads to better evaluations of absorption and metabolism [10,51]. Therefore, these systems enable to observe how the organs are affected by the main compound and by the metabolites that might be generated under physiological conditions [11,67,68].

Multicellular tissues and structures in living organs experience mechanical forces such as blood flow, compression, and tension, that are important for physiological processes [42,44,70,71]. Blood flow is critical for cells maturation and differentiation, as well as for their proper functioning [55,70]. OoC devices enable the reproduction of some forces by modulating the flow characteristics [42,70]. This can be performed by passive manners, when talking of gravity or concentration gradient, or actively, through the integration of micro-pumps together with their respective control and measurement systems [44,54,72,73]. Good simulations of cell migration from one tissue to another, as can be immune cells, or cancer cells in methasteses, are important challenges [7]. Sophisticated microenvironments can also be mimicked by using multilayer technologies, parallel microchannels, cellular spheroids, turning devices, or varying the cellular arrangement pattern, among others [30]. The integration of the support and channel architectures, the 3D cell arrangement and the flow systems enable the appropriate nutrients supply, the waste elimination and the exchange of molecular and analytical signals [13,49,50,52,58,74].

### Measurement systems integration

Although several analytical methods can be coupled to OoC (see Table 2 for some examples), these systems still show lower capacity to adapt to HTS than 2D tissues. OoC will, however, present a higher adaptability as soon as faster and more reliable analytical systems are developed for on-line analysis [4,13,62,71,75].

The integration of sensors in OoC systems provides an additional advantage over animal and human models as electrophysiological signals can be collected and analyzed on real-time[28,58]. This allows the quantification of cellular metabolites and study their evolution in front of external stimuli [53]. Furthermore, the functionality of tissues, cells or even intracellular organelles can be tracked [80].

The main challenge of these measuring systems is to properly assess drug concentration changes and physiological responses by employing low sample quantities at low flows and with high dynamism, in a heterogeneous and complex-composition environment with the presence of salts, sugars and proteins, among others [3,28,53,80]. Aside from the selection of an appropriate analytical technique, an integrated OoC system requires the development of specific computer systems dedicated to analyze and integrate the measured signals [76,83].

## OoC systems production

### 3D Printing

One of the major technological contributions for the development of OoCs is the 3D printing. This technique allows the construction of the desired architecture, communication channels and functional structures with cells and polymeric materials [28,44,57], which makes it possible to control specific conditions by modelling mechanical features [7,28,60]. The support structure is made of polymeric materials that must accomplish three main characteristics: biocompatibility, easy to shaping and transparency. The most commonly used polymer for this purpose is polydimethylsiloxane (PDMS) [1,84]. Four different techniques can be used to form OoC by 3D printing: micro-extrusion, stereolithography, inkjet bioprinting and laser bioprinting [1,28].

In *micro-extrusion*, a continuous flow of small drops of bio-ink (polymer and cell suspension) is generated and pressed out from a small opening whose movement is precisely controlled by a computerized system. Although this technique allows the use of bio-inks with complex compositions and high viscosity, cell viability could be reduced [1,28,57]. Micro-extrusion is one of the most used techniques for the formation of OoC due to its low cost, however, it shows limitations in terms of texture creation and, thus, it is not suitable for the elaboration of complex structures in heterogeneous OoC systems [58].

*Stereolithography* creates a liquid pattern that is later polymerized using a laser or ultraviolet (UV) light. In this technique, cell viability may be affected due to the length of time of the process and the use of intense radiation [28,57,60]. However, by using stereolithography, vascularized channels can be integrated and hydrogels can be introduced in an easier way [57].

In the *inkjet bioprinting* technique, drops of a non-viscous bio-ink are released into a polymeric substrate obtaining a rapid formation of structures by using a low-cost process. There is, however, a lack of precision in the direction of the drop and in the control of the viscosity, which makes the construction of complex designs difficult [28,85].

Finally, *laser bioprinting* uses a laser beam to push a gelled solution of cells onto a substrate. This technique offers high precision in tissue production and a large variety of materials can be used. However, cells viability can be affected by the high temperatures produced by the laser during the long processing time [1,58].

### Cell lines obtention

The correct selection of biological sources for the production of cellular tissues is a key factor to obtain a greater similarity between OcCs and human tissues. The choice of the source will depend on the organ and the genetic background aimed to be studied [2,26,42]. Both primary tissue cells and immortalized lines can be used for these purposes, although the latter may show more difficulties performing the physiological functions of primary cells [42].

**Figure 3.**
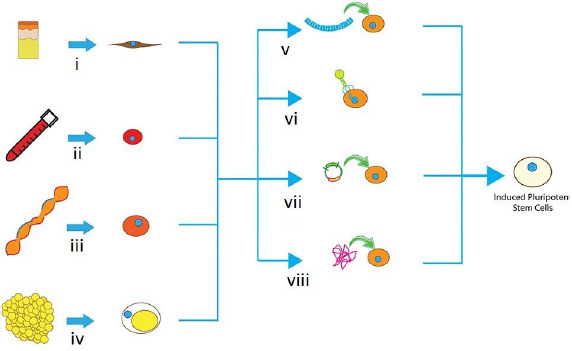
Diagram of iPSC obtainment from **i)** Fibroblasts, keratinocytes or melanocytes obtained in skin biopsy, **ii)** CD34+ from blood samples, **iii)** CD133+ from umbilical cord or **iv)** multipotent cell from adipose tissue; iPSC reprogrammed towards pluripotent cells through different induction mechanisms such as: **v)** microRNA delivery, **vi)** viral transfection, **vii)** integration vectors or **viii)** protein transfection. *Adapted from [40] under terms of the Creative Commons Attribution License. Copyright (2017).*

Numerous authors have cited the use of induced pluripotent stem cells (iPSCs) for regenerative medicine, drug discovery and disease modelling [2,3,5,42,74,86–91]. These cells show an extraordinary organizational capacity that allows them to be modelled to any physiological or pathological condition [2,62,74,86]. Figure 3 shows an outline of iPSCs preparation from various sources by using different reprogramming methods, including CRISP-Cas9 genome editing [92].

The integration of iPSCs in an OoC can be a useful tool for personalized medicine. These kind of cells have a great capacity to induce genetic variability [65,90] and make it possible to execute specific pathological evaluations, which cannot be performed through current alternatives [2,5]. Musah *et al*. established protocols to differentiate iPSCs into mature-functional podocytes and built a human glomerulus in a chip with the aims of nephrotoxicity screening, therapeutic development and the study of kidney development and renal impairment [87]. Smith *et al*. highlighted methods to produce iPSC-derived cardiomyocytes for efficacy/toxicity screening and modelling of cardiac diseases [65]. Moreover, Wang *et al*. developed liver organoids that showed hepatic functions from iPSCs [89]. In another approach, Vatine *et al*. used patient-derived iPSCs from individuals with neurological diseases to simulate the disorder’s specific physiopathology [74].

## Applications

OoCs are currently used in preclinical phases as a complement to animal models and cell cultures [1,3]. 2D models cannot be used for drug evaluation in terms of metabolic activity, efficacy or systemic toxicity [31,42,43,63]. Nevertheless, OoCs have the potential to replace traditional experimental models, as they have shown better *in vivo/in vitro* extrapolation [6,51,93] and potentially represent a more favorable risk/benefit value in drug research and development [18]. These systems present outstanding features such as the chance to simulate dynamic multi-organ environment [1,2,81,94] and to replace the use of humans in some phases of clinical studies [80]. Tables 3 and 4 summarize some examples of the applications of OoC technologies for organs, systems and pathophysiological conditions. The following sections point out additional features for some of the most advanced applications with the greatest impact on academic production.

### Heart-on-a-chip

Cardiotoxicity is one of the most common causes of drug failures in phase I clinical trials [13], highlighting the necessity of more accurate prediction models for a preclinical screening. An interesting number of OoC models simulating the human heart have been developed with different levels of complexity. Its main application is centered on the evaluation of the contractile and rhythmic functions [6,7,13,18,44,122]. The OoC application for heart reproduction can be a powerful tool in the preclinical evaluation of a drug, as it allows to connect the heart with the lungs and the liver and to detect toxic reactions that may be absent when the drug is assessed in an isolated tissue [1,11,65].

**Figure 4.**
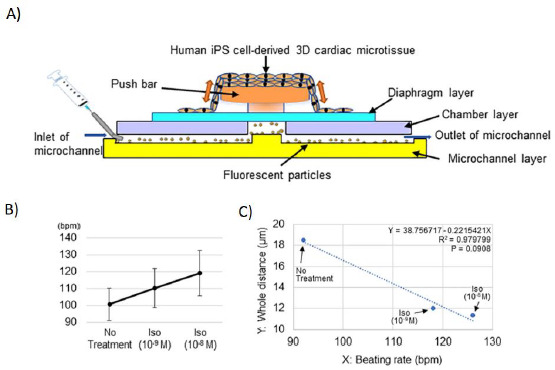
Heart-on-a-chip for the evaluation of physiological parameters. **A)** Device diagram and structure. Graphs **B)** and **C)** respectively show the effect of two different isoprotenol concentrations on the heart rate and the magnitude of contractile displacement, compared to no treatment values. *Adapted from [82] under terms of the Creative Commons Attribution License. Copyright (2020).*

Schroer *et al*. published a work focused on contractile characteristics and parameter determination for personalized cardiac cell tissues [95]. The heart-on-a-chip developed by Abulaiti *et al*, (figure 4) allowed the evaluation of heart tissue function and visualization of cardiac micro-tissue kinetics by monitoring the displacement of fluorescent particles incorporated within one of the device’s compartments through fluorescence microscopy, flow cytometry and high resolution cameras [82].

### Kidney-on-a-chip

The kidney is the main organ for hemofiltration, it is involved in waste excretion and it is indispensable for homeostasis. Therefore, the study of the renal function by using OoC can be a very interesting tool for drug development and for the determination of the systemic safety profile of drugs [2]. The greatest challenge in developing functional kidneys-on-a-chip is the simulation of the tubular structure with a sophisticated control of the physical parameters involved in the glomerular filtration [1,2,87]. The introduction of an on-line renal function detection unit in the OoC allows compounds at very low concentrations to be detected in the system [1].

**Figure 5.**
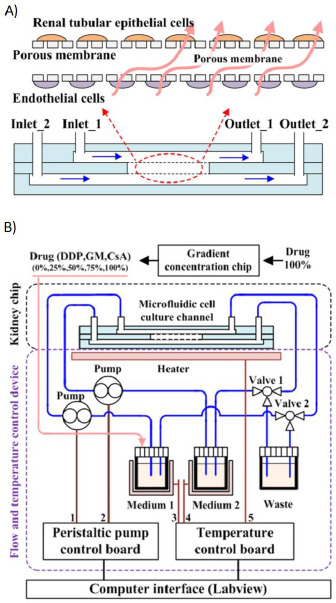
Kidney-on-a-chip device for drug screening and nephrotoxicity assessment. **A)** Schematic representation of the chip’s two chambers. **B)** Diagram of the complete device including: the chip and the temperature and the flow control device. *Adapted from [97] under terms of the Creative Commons Attribution License. Copyright (2020).*

Some examples of this technology can be found in the work of Yin *et al*, who developed a kidney-on-a-chip device for drug screening and nephrotoxicity assessment (figure 5). They achieved tissue differentiation and functionality by growing the two cell cultures on opposite membranes, and evaluated the cell growth and viability using fluorescent markers [97]. On the other hand, Jang *et al*. built a system for drug transport and nephrotoxicity assessment in preclinical phases that mimicked key functions of the human kidney proximal tubule. The chips architecture exposed cell layers to a fluid shear stress similar of that found in living kidney tubules, resulting in an enhanced epithelial cell polarization, differentiation and function compared to traditional Transwell culture system [98]. Wang *et al*. developed a kidney-on-a-chip for the study of virus pathogenic mechanisms inducing renal infections, which allowed in-line cell characterization using confocal imaging and scanning electron microscope imaging [45].

### Lung-on-a-chip

**Figure 6.**
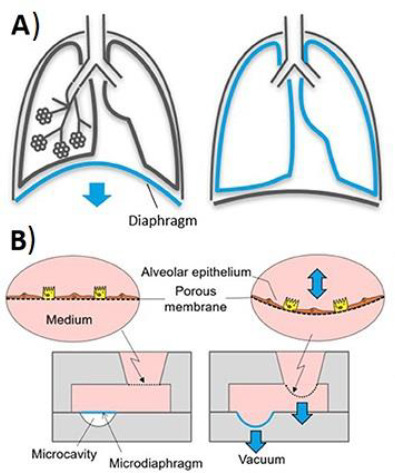
Mimicking of breathing mechanics. Schematic representation of **A)** the movement of the diaphragm leading to the lungs expansion during inhalation and **B)** the imitation of this motion by a mechanical microdiaphragm used in lung-on-a-chip devices*. Reproduced from [100] under terms of the Creative Commons Attribution License. Copyright (2019)*

Modern OoC systems are inspired by the pioneering development of a lung-on-a-chip. The main challenges for an accurate lung function are the generation of biomechanical ventilation, reproduction of lung mucosa and the capability to overcome the possible prothrombotic interference and the rigidity of the simulated lung tissue [1,13]. Stucki *et al*. developed a system that enables the imitation of the alveolar parenchyma and that can produce a mechanical stretching that mimics the breathing mechanism using a pneumatic micro-diaphragm. They demonstrated that the mechanical stress affects the epithelial barrier permeability [99], which id something to consider when manufacturing other lung models reproducing somehow this kind of mechanical stress. Felder *et al*. studied wound healing recreating an alveolar epithelium on a chip also including mechanical strain with the incorporation of an ultra-thin elastic membrane (figure 6). This device used immunofluorescence microscopy and scanning electron microscopy for the evaluation of test outcomes [100]. The lung-on-a-chip developed by Asad *et al.* incorporated on line detection of pH and trans-epithelial electrical impedance, as well as a microscope that allows visual monitoring due to the chip’s transparency [101].

### Gut-on-a-chip

The small intestine has important functions associated with drug absorption and the immune system [2,7]. Applications for OoC-gut models include pharmacodynamics, pharmacokinetics and physiological studies, and are frequently linked with the use of CaCo2 cells. These gut-on-a-chip models enable to investigate the way how the tissue-microbiota interrelationship can affect the nutrient absorption in the gut, and allow the study of the signaling between gastric and intestinal cells [1,54,72,103]. OoC-gut models have also been used to evaluate the mucus formation, accumulation and impact on intestinal functionality [31,72].

**Figure 7.**
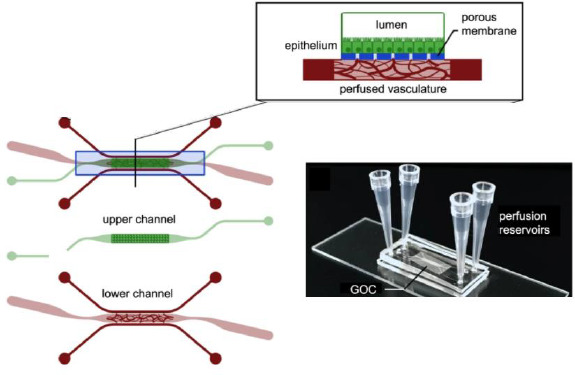
Gut-on-a-chip (GOC) formed by two channels separated by a thin membrane with perfusion vasculature on the lower channel*. Adapted from [105] under terms of the Creative Commons Attribution License. Copyright (2020).*

Peters *et al*. highlighted the importance of *in vitro* testing to assess gastrointestinal safety [54]. In another approach, De Haan *et al*. monitored enzymatic digestion of milk using a microfluidic chip and demonstrated the importance of adjusting and correlating digestion times at physiological conditions on OoC [69]. Sailer *et al.* built a gut-on-a-chip from patient-derived intestinal subepithelial myofibroblasts, which incorporated growth factors and controlled physiological conditions that showed angiogenic properties under simulated perfusion (figure 7) [105]. The gut-on-a-chip also can be found coupled with other organs, such as the liver, which allows to study the absorption and metabolism of drugs [51].

### Liver-on-a-chip

The liver plays a crucial role in the drug metabolism and the detoxification of the body. Therefore, it is another important organ to take into consideration during the development of new drugs and their effects in the other parts of the system [2,11,21,89]. Liver cells grown under 3D conditions are more viable, functional and reproducible than those cultured in 2D [2,108]. Liver-on-a-chip models associated with other tissues or organs, such as the intestine, may allow to obtain first-step metabolism profiles closer to those that could be acquired through traditional cell cultures [1,3,89].

**Figure 8.**
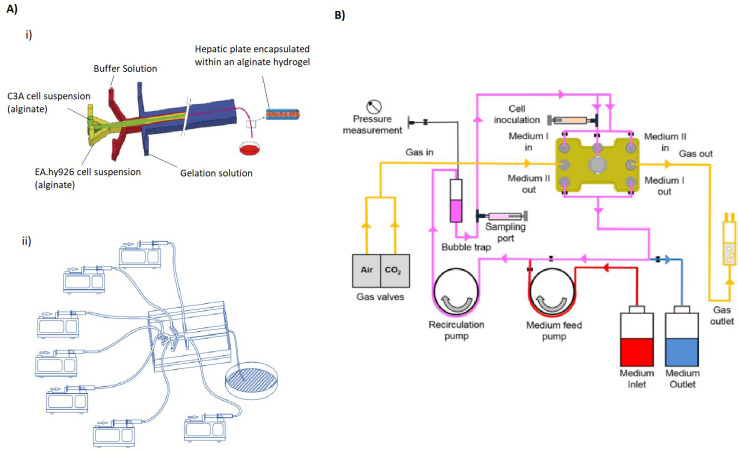
Schematic examples of liver-on-a-chip devices. **A)** Multichannel OoC. Representation of a **i)** microfluidic device with several channels of different compositions for the formation of encapsulated hepatic plate structures, and a **ii)** system of syringe pumps supplying the microfluidic channels of the OoC with solutions. Adapted from [43] under terms of the Creative Commons Attribution License. Copyright (2020). **B)** Diagram of a pressure-driven flow control system of a 3D liver bioreactor for hepatotoxicity testing under perfusion conditions. *Adapted from [107] under terms of the Creative Commons Attribution License. Copyright (2018).*

Jia *et al*. described a 3D hepatic system for liver injury studies by controlling hepatocyte viability, function, morphology and polarity, and thus, maintaining the tissue morphology, mRNA expression, bile secretion and a better cell viability compared to other types of cell cultures. They controlled the morphology encapsulating the cell culture inside a alginate hydrogel (figure 8A) [43]. Freyer *et al*. built an OoC model for hepatotoxicity testing, highlighting the importance of the control and monitoring of chemical physiological parameters (such as lactate production and ammonia release) (figure 8B). They validated the OoC by performing a paracetamol hepatotoxicity determination, which included a histological and immunohistochemical evaluation of the damage. They also stablished the need of modifications of the OoC to include non-parenchymal cell types at physiological ratios and the creation of defined oxygen gradients in order to increase the system’s sensibility for further toxicity studies [107].

On another approach, Kostrzewski *et al*. prepared a 3D culture on a plate from primary cells that showed positive progression on fatty liver recovery by using known anti-steatosis compounds. This model showed adipokine expression and metabolic responses similar to those seen in clinical non-alcoholic fatty liver disease. The chip is not designed for on-line quantifications and most of the analytical determinations had to be made after removing the microtissues from the chip [108]. As well as the heart or the intestine, the liver has been studied in multiorgan chips to investigate systemic effects, absorption and metabolism [51,67,68].

### Central nervous system-, brain- or blood brain barrier (BBB)-on-a-chip

It is possible to simulate physiological and pathological mechanisms of the central nervous system (CNS) in an OoC [2,88]. These models can be very useful considering the complexity of the human brain, which makes it difficult to study the CNS in animal models or 2D cultures [13].The blood-brain barrier plays a vital role in homeostasis and it is the main limitation for the administration of drugs in the brain [109]. The development of integrated systems reproducing the barrier properties and modelling inter-individual variation [2,15,74] makes it possible to assess whether a drug designed to treat a neuro-related disease can actually reach its therapeutic target [13,78].

**Figure 9.**
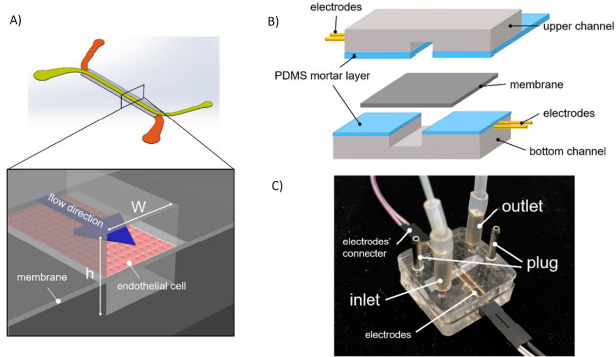
BBB-on-a-chip with microelectrodes incorporated. **A)** Diagram of the main channel. **B)** Diagram of the electrodes and the chip structure. **C)** Photograph of the integrated system. *Adapted from [123] under terms of the Creative Commons Attribution License. Copyright (2020).*

Adriani *et al*. were able to develop a chip with neurons and astrocytes that showed adequate morphology, growth and vascular function, identifying the opportunity to include other types of cells such as pericytes and microglia, which increased the model’s potential to be used for neurovascular studies and too assess drug effects on brain neural function [48]. The brain endothelium created from primary patient cells by Vatine *et al*. reproduced complex BBB functions with the potential applications of drug screening and personalized medicine, using immunocytochemistry, confocal microscopy, calcium imaging and mass chromatography as analytical methods, among others [74]. Tu *et al.* developed a BBB system that incorporated transendothelial electrical resistance (TEER) micro-electrodes to monitor cell growth and response in the presence of drugs (figure 9) [123].

### Inflammation-on-a-chip, or immune system-on-a-chip

**Figure 10.**
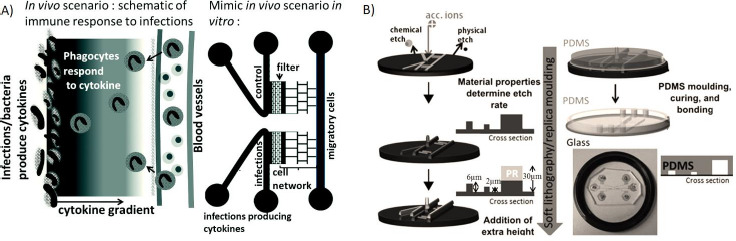
Microfluidic device allowing cell migration. A) Simulation of an *in vivo* scenario with the chip construction. **B)** Schematic of the device’s manufacture by soft lithography. *Reproduced from [131] with permission from The Royal Society of Chemistry. Copyright (2015).*

OoC can also reproduce complex physiological events or multi-organ functions [7,128]. The development of the inflammatory system-on-a-chip enables a better understanding of how immune system cells synchronize their activities to fight a disease. The evaluation of the coordinated cellular activities related to inflammation could have interesting biological and clinical applications [128]. Autoimmune diseases or immunity generated by vaccines can also be assessed by OoC systems [50,71,102]. Sharifi *et al*. developed a system on a chip for modeling the immune response cascade of events during immune cell response to implants [130], and Goyal *et al*. described a microfluidic chip for evaluation of vaccine response that was able to obtain antibodies and can be useful for immunotherapy candidate selection [130]. Gopalakrishnan *et al.* worked on a real-time continuous monitoring chip (using microscopy and fluorescence) for studying cellular migration and organization during inflammatory processes in response to simulated cytokine gradients (figure 10) [131].

### Cancer-on-a-chip

Cancer models in mice have resulted in countless treatments to cure cancer in the animals, although they present an alarming difficulty to transfer these results to humans. OoC systems provide the possibility to study the physio-pathological mechanisms and the resistance reactions in an environment almost identical not only to human disease but to the specific individual. Therefore, these systems can open the door to many treatments against cancer [13,14].

**Figure 11.**
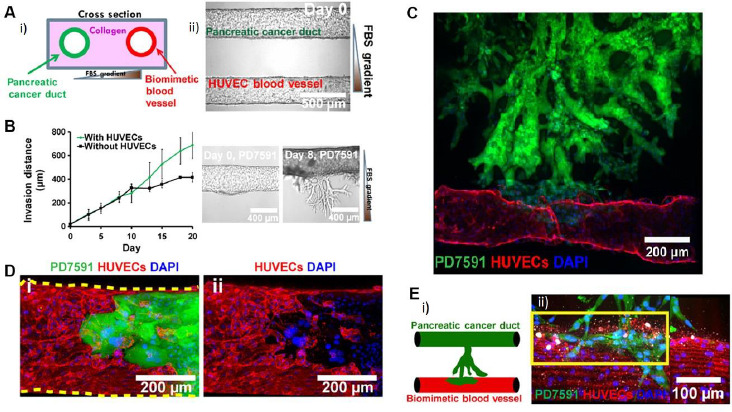
Pancreatic-cancer-on-a-chip. **A) i**: image of a pancreatic duct (seeded with pancreatic cancer cells) and a blood vessel (seeded with endothelial cells) nested within a collagen matrix that are shown on the right image; **ii:** representation of the cross section of the right image. **B)** Invasion distance that cancer cells traverse within the collagen matrix as a function of time, with and without the addition of human umbilical vein endothelial cells (HUVECs). **C)** and **D)** Invasion of tumoral cells (green) into the blood vessel (red). **E) i**: invasion of the blood vessel by cancer cells, inducing to the apoptosis (marked in white in **ii**) of endothelial cells. *Modified from [125] under terms of the Creative Commons Attribution License. Copyright (2019).*

Scientists have been able to modulate through OoC devices phenomena such as metastatic cells migration, tumor growth or even drug resistance [124,132]. Moreover, potential therapeutic targets and possible chemical markers have been identified in preliminary research based on OoC [15]. Nguyen *et al*. used an OoC model to demonstrate pathogenic mechanisms involved with the endothelial changes observed in pancreatic cancer, as well as to identify chemical mediators of these phenomena (figure 11) [125]. Shuford *et al*. used primary patient tissue on a chip for the prediction of patient-specific response before clinical treatment in ovarian cancer [126]. Choi *et al*. designed a device with the microarchitecture of breast carcinoma to be used as drug screening platform. They validated the screening capacity using it to evaluate efficacy and toxicity of paclitaxel [127].

## Body-on-a-chip or human-on-a-chip for ADME evaluation and preclinical development

OoCs can assembly multiple tissues and simulate the systematic functionality of the human body. As long as each organ shows the most relevant functions associated with the pharmacokinetic phenomena to be studied, these systems have the potential to determine the pharmacokinetic profile of drugs [7,10,13,42,50,102]. The processes of absorption, distribution, excretion and metabolism (ADME) can be simulated from various routes of administration and, moreover, mathematical models can be applied to make real-body predictions [10,27,51,66,132,133]. This approach has been used to evaluate the ADME profile of drugs and the appearance of physiological events that cause adverse effects in devices of up to 20 different tissues [7,10,32]. Furthermore, they can also be used to assess a compound effectiveness [13,133]. De Melo *et al*. developed a heart-liver-skin device for the systemic evaluation of drugs administrated topically and for the comparison of acute and systemic toxicity (Figure 2B) [67].

The system developed by Rajan *et al*. including tissues from the brain, liver, lung, heart and endothelium showed metabolizing capacity for prodrug transformation, as well as representativity of cardiac toxicity [10]. Marin *et al*. studied acetaminophen absorption and metabolism with an intestine/liver micro-physiological system demonstrating the potential of these kind of systems for pharmacokinetic profiling of drug substances [51]. The cardiotoxicity of the main compound and the metabolites was evaluated with an OoC device by Oleaga *et al*., who developed a model that can be used in preclinical evaluation and for chronic studies [11]. Maschmeyer *et al*. proposed one of the first devices to integrate intestine, liver, skin and kidney organoids for ADME profiling, and repeated dose systemic toxicity testing of drug candidates, which was sustainable throughout 28 days [134]. The system of fluidically coupled vascularized chips proposed by Herland *et al.* allowed modeling the pharmacokinetics of orally and intravenous administered drugs. Their arrangement was able to couple with robotic pumps, chips that represent the gut, liver, kidney, and bone marrow, and presented an arteriovenous fluid-mixing reservoir. They obtained pharmacokinetic results similar to previously reported clinical data [135].

One of the most important challenges in the body-on-a-chip development is the proportional replication of organs in terms of size, blood flow and metabolic rates. However, in many cases, these proportions are still unknown for the human body [7]. Another challenge of these OoC devices is that they represent a small number of cells, and their intercommunication substances undergo under significant dilution conditions [42].

Nearly a 70 % of drug withdrawals correspond to hepato-cardio toxicity, therefore, it is believed that a system that included at least those organs would be enough for a preclinical safety screening [136]. OoCs could be used in the future for clinical studies by introducing specific variables needed according to personalized groups of patients. Moreover, this might allow studies of pathologies for which there are usually not enough candidates for a representative clinical trial [13]. The use of OoC systems in the development of new drugs would enable designs based on better physiological understanding and better candidate screening [86]. This would lead to an increase in drug development efficiency by reducing costs (up to 25 %) and time for the experimental phases [9,18,29], lowering the risk for patients and raising the investment in new drugs production [13]. This could increase the number of new discoveries and reduce their prices, benefiting, therefore, the health systems at various levels [9,18,20,71].

## Challenges and perspectives

In order to exploit the full potential of OoC systems and make them become a validated alternative, several physical, physiological and regulatory challenges must be overcome. The first step to obtain sophisticated and accurate OoC with a minimum functional unit [13,133] is to have a deep knowledge of the organ’s physiology, the cellular metabolism and all pathological mechanisms that can affect the organ [31,44,50,53]. Table 5 shows the main challenges or advances required in the manufacture of OoC systems for some specific organs.

As a field that influences a wide spectrum of disciplines [22], OoCs require significant advances in the availability and the implementation of the newest trends from the scientific areas related to their design, development, application and production. Overcoming the individual challenges is the key for translating OoC to cost-effective and sustainable implementation. We briefly introduce some of those relevant challenges, however, more insight into each research area can be found on specific reviews developed for this purpose, which can be found elsewhere [33–41,137,138].

### Challenges in fluid mechanics

Microfluidics are essential for the reproduction of the organ’s conditions in an OoC. The use of external systems is critical [73], as well as the 3D arrangement of the microchannels. Many of the current devices present a gravity-mediated flow distribution [7,11], however, the development of better microfluidic control systems is essential to improve the reproduction and to control the microflows that generate more bio-relevant conditions within the chips [42,73].

The development of microfluidic is, perhaps, the most important driver in the progress to more complex, robust, significant and scalable OoCs. For example, HTS using OoC devices requires improvements in microfluidics that allow a better controlled perfusion, sampling and cell injection [18,139], while on the other hand, stem cell’s experimentation, production and analysis requires high level of microenvironmental control [140]. Chips with integrated micro pumps and, with structural and mechanical considerations that allow cell circulation and passive flow control are some of the microfluidic topics with more progress in the past years [41].

### Challenges on cell availability

Physiological reproducibility of OoC mainly depends on the cells that are employed to build them. Cell availability challenges in OoC are the same restrictions than those related to cellular tissues: limited access to primary cells and possible dysfunction of immortalized cells [7,42]. To have different cell culture types in the same environment brings to an extra difficulty, which is shared with regenerative medicine techniques [28,90]. Obtaining reproducible tissues, suitable for application in OoC systems, requires the development of more and better techniques for the culture, maturation and differentiation of pluripotent cells, as well as the standardization of the obtention of protocols [5,55,90].

Major breakthroughs in this field include relevant experimentation on novel and standardized methods to obtain and differentiate stem cells into functional organ models [141–143], and the continuous efforts for the development of a blood substitute, a representative universal cell medium for OoC [41].

### Challenges in materials engineering

In this area it is required to develop materials that accomplish all –or most of– the application criteria in an OoC, such as plasticity, elasticity, transparency, biocompatibility or versatility [7,63]. These materials must be inert to the drugs to evaluate [44,75] and exhibit easy printing, while achieving adequate mimicry with living tissues [28]. The materials that are currently used do not meet all the requirements simultaneously. For example, PDMS is one of the most widely used materials to manufacture OoC due to its versatility and biocompatibility, nevertheless, it has shown a tendency to absorb small molecules, such as many of the drugs evaluated [10,18,30,144]. Therefore, the discovery of intelligent materials that can change their shape and reactivity in front of different stimuli, known as four-dimensional printing, is a promising strategy for the future OoC [58]. Recent advances include the introduction of a variety of biocompatible and thermoplastic materials that are presumably more suitable for large scale manufacturing processes [41].

### Challenges in measurement systems

The evolution of OoC requires an improvement in the development of specific electrodes and/or biosensors [7,28] with high performance, high sensitivity and high specificity [18,53,80]. The high sensitivity is required due to the low amount of metabolic secretions [3]. Moreover, it must be possible to measure the tissue response at many other levels, as well as the concentration variations of the specific substance [42]. Therefore, OoC systems must have the ability to detect small non-lethal changes that affect functionality at the cellular level [7,41]. More feasible, adaptable to HTS and scalable bio-assays also require microfluid regulators such as valves, pumps, mixers and other functional elements to get cell perfusion with fresh media and assay reagents [139]. These systems must allow non-invasive parallel measurements, automation and flexibility in order to fit HTS programs used by Big Pharma companies to evaluate their compound libraries [4,18,29,41,75].

**Figure 12.**
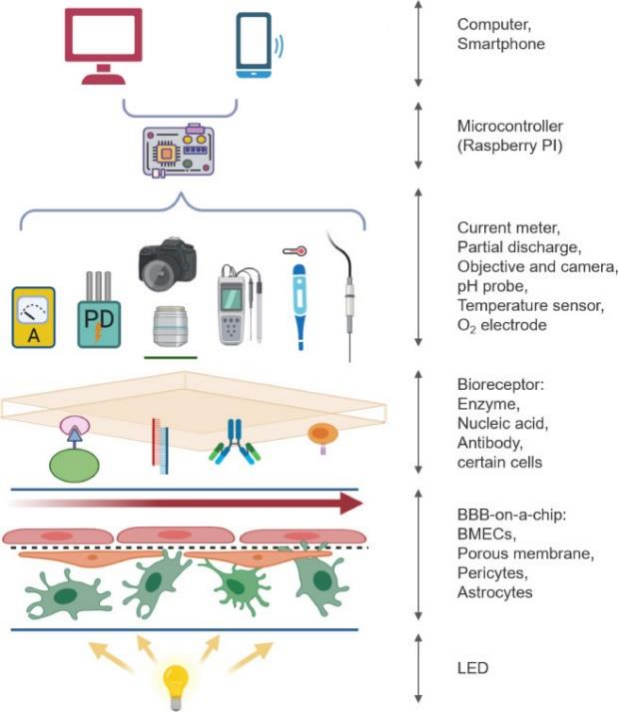
Biosensors and sensors on BBB-on-a-chip transducing signals to a PC or mobile interface. *Reproduced from [146] under terms of the Creative Commons Attribution License. Copyright (2019).*

Commercially available micro-sensors of thermos responsive materials and electrodes, as well as optical methods for pH and oxygen determination, have been successfully incorporated to OoC devices, [41] in addition to several prototypes of organic biotransistors [145]. In the particular case of a BBB-on-a-chip, TEER has become the most popular online sense method. But other alternatives such as physical sensors, with the ability of measuring small concentrations of metabolites, and a variety of biosensors, bioreceptors that generate an electric signal after binding a biological target, have been proposed (figure 12) [146]. Software-driven analyses of optical recordings have been described for several cellular functions such as the heart rate [82], which can be used to evaluate other related muscular functions.

### Challenges on industrial scale production

There are many OoC prototypes that have been presented only as a pilot test at laboratory level. The success of an OoC system in solving real problems depends on how rigorously it fits the real model, but furthermore, on how easily it can be transferred to industrial production. In many cases, the OoC prototype is obtained through an effective 3D printing technique, but it is inefficient for large scale due to the low speed at which the structures are created [7,11,28].

Low cost or reusable materials could lead to a more widespread use of OoC in early stages of drug discovery [143]. Massive parallelization and automation of OoC systems are among the biggest challenges to overcome in order to meet industrial requirements of HTS [18]. The work of Ramadan and Zourob [137] contains a comprehensive review not only of the main challenges, but of specific strategies to solve them, trends and examples of the main industrialization activities of some of the developing OoC systems.

The more sophisticated an OoC becomes, the more it needs to be controlled, which increases its cost and limits its usege. To ensure reproducibility of OoCs [49] specific tests must be developed in parallel for the quality control of the devices [133]. Besides any limitation, the number of commercially available OoC devices, new start-up companies and patent applications have considerably increased these last years [136].

### OoC validation

The validation of OoC systems is particularly important for regulatory agencies to accept them as clinical and experimental alternatives, especially in toxicity trials [13,42,43]. So far, many OoC developments correspond to isolated publications with a lack of standardized protocols for operation, data collection and analyses [63]. Direct comparisons require specific clinical studies and long-term gathered data. Thus, many OoC system improvements are performed in collaboration between academia, industry and regulatory authorities [7,50]. Regulatory agencies, academia and several companies have been conducting efforts to establish industrial standards and validation guidelines for OoC technologies [136]. The replication of already-conducted trials executed with approved products and the comparison of their results with the results obtained with approved assays is one way of achieving the acceptance of OoC systems as alternative methods for pre-clinical –or even clinical– studies [7,136]. OoC could be used to replicate cases of clinical studies in which severe adverse events were not detected pre-clinical stages and drugs had to be withdrawn from the market, therefore, it could be determined if this technology has a greater or equal predictive capacity [44].

## Conclusions

There is growing evidence that OoC systems are a novel tool that will facilitate the search, development and evaluation of new drugs. These systems show characteristics of reproducibility, adaptability and versatility that make them a potential alternative to the use of experimental animals.

OoCs are an evolution of 2D cell cultures, thus maintaining some of the characteristics of cell tissue evaluations. However, these new systems overcome many of the conventional cell cultures weaknesses, such as the poor extrapolation to an entire organism. OoC simulate the real microenvironment and the key functional aspects of organs at a microscopic scale. This enables the study of drugs as well as the investigation of physiological and physio-pathological phenomena.

The construction of OoC requires a deep understanding of the real functionality of the tissue/organ to be studied and mimicked. In many cases, this knowledge is limited, thus leading to a synergy between the research in physiology and the development of OoCs. These systems could –scientifically and financially– promote the development of new drugs. Risk reduction could revolutionize the way big pharmaceutical companies focus their development efforts, as well as the approach of how clinical and preclinical studies are conducted.

OoC's progress efforts must converge on the development of systems with enough representativeness, reproducibility, scalability and suitability for HTS, which maintain at the same time reasonable production and implementations costs. Although science is far from being able to mimic at 100 % the human body in a laboratory, any advance will increase the predictive potential of OoC systems and will provide innumerable benefits for new drugs development.

## Figures and Tables

**Table 1.** Main comparative characteristics of traditional preclinical evaluation models and OoC. Modified from [5].

Characteristics	Animal	2D	OoC
Target tissue’s representativeness	Low	Low	High
Ethical implications	Medium	Low	Low
Associated costs	High	Low	Low
Viability maintenance	Medium	High	High
High throughput analysis aptitude	Low	High	Medium
Full body representativeness	High	Low	Medium

**Table 2.** Main analytical techniques reported for on-line analysis of OoC.

Analytical technique	References
Fluorescence	[24,53,76,77][78]
Electrochemical detection	[24,79,80]
Microscopy	[48,80,81]
Chromatography	[3,80]
Electrophoresis and Magnetophoresis	[3,75]
High definition cameras	[24]
Color change (pH)	[28]
Light scattering	[76]
Ionization Spectroscopy	[80]
Filtration	[75]
Immuno-agglutination	[76]
Potentiometry	[24]
Trans-epithelial resistance	[24]
Acoustic techniques	[75]
Photoacoustic Tomography	[44]
Force transducers	[24]
Flow cytometry	[82]

**Table 3.** Overview of some functionalities and applications of organs or systems on a chip. Modified from [1,2].

Organ or System	Main components of the OoC functionality	Preclinical application	References
**Heart**	- Contractible 3D conformation. - Incorporation of structural proteins. - Electrical stimulation. - Regulated mitochondrial distribution.	- Simulation of Frank-Starling mechanics in cardiomyocytes. - Demonstration of auxotonic contractions. - Cardiotoxicity tests. - Study of physiological phenomena involved in cardiac functioning.	[2][4][6][7][11][18] [21] [44][71][70] [65] [82] [84][95] [96]
**Kidney**	- Barriers formation. - Permeability modulation with different types of proteins. - Generation of structural proteins. - Functional assessment under mechanical stress conditions. - Separation of tubular flow and interstitial fluids.	- Selective filtration in kidney models. - Simulation of type II diabetes mellitus nephropathy. - Evaluation of drug-induced nephrotoxicity. - Research on the physiological role of specific proteins. - Study of viral infections.	[1][2][4][13] [21] [45][87][75][84] [97][98]
**Lung**	- Simulation of the alveolus/capillary interface. - Modulation of cell permeability. - Presence of intercellular proteins. - Simulation of air-water interfaces with or without the presence of airflow.	- Breathing simulation using cyclic mechanical stress. - Evaluation of resistance mechanisms in lung cancer. - Lung proteins study. - Metabolism of drugs. - Systemic toxicity studies.	[1][13][18][21][29] [42][52][54][71] [99][100] [101]
**Gut**	- Differentiation of the intestinal epithelium. - Reproduction of peristalsis. - Incorporation of extracellular proteins. - Reproduction of intracellular and paracellular transport.	- Microbiota interactions studies. - Pathogenic bacteria models. - Study of viral infections. - Simulation of barrier function loss. - Drug absorption and metabolism evaluation.	[1][2][4][7][8][13] [31][51][52][69] [72][102][103] [104] [105][106]
**Liver**	- 3D cultures with specific liver substructures. - Kupffer cell incorporation. - Incorporation of structural proteins. - Secretion and production of high levels of urea and albumin. - Enzymatic activity. - Production of bile and formation of gallbladder.	- Drug metabolism studies. - Hepatotoxic effect of drugs and toxicity effects on other organs (including the influence of liver metabolites). - Glycogenesis studies (and inhibition by drugs). - Fatty liver drug development.	[2][4][11][13][43] [51][67][68][71] [89] [99][104][107] [108]
**Brain/Central Nervous System**	- Blood-brain barrier (BBB) formation in the presence of astrocytes and neurons, and with modulated cell permeability. - Formation of tubular vessels. - Structural protein incorporation. - Brain folding. - Replica*tion of the neurovascular unit.*	- Study of neurodegenerative diseases. - Inflammation studies by lipopolysaccharide endotoxins or others. - Study of the influence of drugs in the prenatal period. - Determination of permeability of drugs and nanoparticles through BBB.	[1][2][4][21][48] [49][64][49][74] [75][88][109]
**Placenta**	- Formation of the placental barrier. - Permeability modulation for high molecular weight proteins. - Division of fetal and maternal chambers with fluid flow. - Transfer of nutrients and glucose to fetal compartments. - Evaluation of placental responses.	- Simulation of drug transport across the placenta using cancer-derived cells. - Study of the influence of drugs in the prenatal period.	[1][21][110][111]
**Adipose tissue**	- Creation of adipose spheres that simulate adipose tissue in vivo. - Vascular-adipose tissue interface. - Fatty acid absorption.	- Glucose uptake studies. - Obesity models. - Development of cell retention methods.	[1][96][102]
**Retina**	- Retinal pigment formation. - Development of the epithelium-choroid structure. - Presence of structural proteins.	- Pathologies models. - Reproduction of imaging processes.	[1][62][112][113]
**Muscle**	- Contractile units through chemical or electrical stimuli.	- Evaluation of the effect and toxicity of drugs. - Study of degenerative diseases and muscle physiology.	[2][114][115]
**Others**	Immune system [50,71], bone [116,117], breast tissue [118,119], pancreas [120,121], bladder, reproductive tract [104], skin [13].		

**Table 4.** Main pathological conditions simulated in OoC systems

Pathology	References
Cancer	[13–15,23,42,50,63,122,124–127]
Inflammation	[7,128–130]
Epilepsy	[44] [64]
Alzheimer's disease	[64]
Musculoskeletal abnormalities	[23]
Arthritis	[23]
Arrhythmias	[44]
Diabetes	[102]
Hematological diseases	[23]
Pneumonia	[44]
Parkinson	[64]
Thrombosis	[23]

**Table 5.** Main challenges for the improvement of some specific organ-on-a-chip systems: Modified from [1,7].

Organ	Main challenges
Kidney	Formation of glomeruli with podocytes and entangled proximal tubules.
Lung	Generation of biomechanical ventilation without assistance. Further studies with inflammatory and immunological processes and tumors.
Heart	Establishment of validated studies for reproducible cardiotoxicity with various drugs.
Gut	Deepening knowledge of mesenchymal-epithelial interactions.
Placenta	Modelling with primary cells.
Adipose tissue	More knowledge of the functioning of brown fat in adipose tissue.
Brain	Standardization and reproducibility of brain organoids.
